# Effects of Walking and Barre Exercise on CES-D, Stress Hormones, hs-CRP, and Immunoglobulins in Elderly Women

**DOI:** 10.3390/jcm14051777

**Published:** 2025-03-06

**Authors:** Min-Kyo Kim, Su-Han Koh, Tae-Kyu Kim

**Affiliations:** Department of Physical Education, Pusan National University, 2 Busandaehak-ro 63beon-gil, Geumjeong-gu, Busan 46241, Republic of Korea; kmk800108@naver.com

**Keywords:** elderly women, walking, barre, stress, depression, immunoglobulins

## Abstract

**Objectives:** This study explored the impact of a 12-week walking and barre exercise program on depression levels (CES-D), stress hormones (dopamine, epinephrine, norepinephrine, cortisol, serotonin), high-sensitivity C-reactive protein (hs-CRP), and immunoglobulins (IgA, IgG, IgM) in women aged 65 and above. **Methods:** Twenty-seven participants were divided into a walking exercise group (WG), barre exercise group (BG) and control group (CG), each comprising nine individuals. Sessions lasted 50 min, thrice weekly, with intensity progressing every 4 weeks. Two-way repeated measures of ANOVA assessed time–group interactions and main effects, and paired *t*-tests and one-way ANOVA were used post hoc with significance set at 0.05. **Results:** The CES-D scores demonstrated significant interaction (*p* < 0.001), decreasing significantly in the BG and WG (*p* < 0.01) while rising in the CG (*p* < 0.05). Post hoc analysis revealed significantly lower depression levels in the BG compared to the CG (*p* < 0.01). Stress hormones epinephrine and cortisol showed a main effect of time (*p* < 0.05), with epinephrine increasing significantly in the BG (*p* < 0.05) and cortisol decreasing in the BG and WG (*p* < 0.05). An interaction effect emerged in hs-CRP levels (*p* < 0.05), while IgA and IgM displayed interaction effects (*p* < 0.05 and *p* < 0.01, respectively), both significantly increasing in the BG (*p* < 0.05). **Conclusions:** In summary, regular exercise positively impacted depression, stress hormones, and immune functions in older women.

## 1. Introduction

Statistics show that depression prevalence in South Korea is 36.8%, indicating that 4 in 10 individuals suffer from both major depression and depressive symptoms [1]. The potential risks of depression include insufficient physical activity and reduced physical functions due to aging [2].

The psychological determinants of depression levels in older adults are reciprocal with stress [3,4]. A state of balance can be maintained through homeostasis in the human body when a certain stress level persists. Nevertheless, chronic stress suppresses the autonomic nervous system activities and breaks the body’s balance owing to the detrimental effect of prolonged exposure to stress hormones, which negatively affect aging [5].

Stress hormones play a crucial role in maintaining homeostasis in the human body. Cortisol is a hormone secreted by the adrenal cortex in response to stress, and the secretion is regulated by adrenocorticotropic hormone (ACTH). An abnormally high or low cortisol level interferes with the physical rhythm, leading to continuous stress, and impacts physical and psychological disorders [6].

Concurrently, physical stimulation of the human body, such as exercise, induces changes in neurotransmitter secretion, including serotonin. Notably, such changes are associated with energy metabolism, homeostasis maintenance in the body, and psychological mood [7]. An imbalance in serotonin function could cause depressive symptoms, a topic that has been extensively researched in numerous studies [8,9].

Additionally, high-sensitivity C-reactive protein (hs-CRP) is a well-known predictor of cardiovascular disease and is closely associated with coronary artery disease caused by stress and other diseases [10,11,12]. Various changes occur in the immune system owing to aging. Similarly, immunoglobulins undergo changes in correspondence with the aging immune system. Exercise reduces inflammation by controlling and offsetting immune system changes while enhancing immunosurveillance to protect the body against external stress [13].

Consequently, physical or psychological stress is a risk factor for chronic disease, and preventing and managing stress is critical in older adults. With recent advanced lifestyle trends, the demand for new exercise programs has increased; however, the participation of older adults who are ≥65 years old continues to rely heavily on walking exercise (37.3%) [14]. Meanwhile, barre is a whole-body exercise that engages both the upper and lower extremities, and performing just the basic motions could enhance the functional fitness factors older adults require [15]. The term of ‘Barre’ is originated from the hand bar in ballet practice, and this program was initially developed by Lilian Gehring who was a ballet dancer from Germany. As barre is a leisure activity involving several individuals practicing together and performing esthetic motions with music, it may also provide a transcendent experience to the participants. Also, in recent years, this barre exercise has received attention from Hollywood, because this elegant and powerful program combines well with ballet and Pilates, effectively training the whole body. Compared to walking, barre has higher posture stability, and this is because it consists of movements that can reduce the risk of falling for the elderly using the bar used in ballet practice. In addition, we think that when performing these stable movements, the elderly can focus more on their postures, so they can increase the effectiveness of the exercise.

Therefore, this study aimed to investigate the effects of 12-week walking and barre exercises on Center for Epidemiologic Studies Depression (CES-D), stress hormone (dopamine, epinephrine, norepinephrine, cortisol, serotonin), hs-CRP, and Ig (IgA, IgG, IgM) levels in older women aged ≥65 years to let older adults recognize the importance and necessity of exercise in improving physical and mental health and to enhance the quality of life in senescence through the healthy living of the older individuals.

## 2. Materials and Methods

### 2.1. Participants

In this study, the participants were older women aged ≥65 years residing in a metropolitan city in South Korea. The recruitment of subjects was only for those who showed voluntary participation; a notice was attached to a local bulletin board. In compliance with The Belmont Report, this study’s purpose and procedures were fully explained to participants, and voluntary written consent was obtained. This study was approved by Pusan National University Institutional Review Board (PNU IRB/2020_129_HR). Participants were selected from those without experience with relevant drug therapy or participating in a regular exercise program (including barre exercise) in the past 6 months and those without experience visiting a relevant hospital for drug therapy or a musculoskeletal injury that could limit the performance of the barre exercise program. In particular, 27 participants were randomly assigned by drawing lots and then divided into a walking exercise group (WG, *n* = 9), barre exercise group (BG, *n* = 9), and control group (CG, *n* = 9). These sample sizes were calculated with the G*power program 3.1. We set the effect size *f* = 0.27, group number *n* = 3, repeated measures *n* = 2, power (1-β err prob) at 0.8, and significant level at 0.5, and a total of 27 participants were calculated. Table 1 indicates the physical characteristics of the participants.

**Figure 1:**
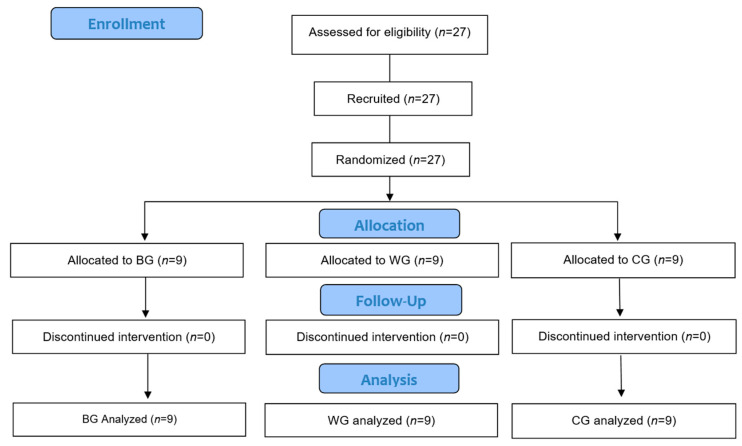
Study flow chart based on Consort 2010 flow Chart Diagram.

Figure 1 shows the study flow chart based on the Consort 2010 Flow Chart Diagram. As a criterion for participation, subjects with no experience participating in a regular exercise program for the last 6 months were selected. As for the exclusion criteria, subjects with muscle and skeletal injuries that may limit their performance in the exercise program and those with anemia and diabetes-related diseases were excluded because the subjects of the study were elderly and blood was collected after fasting.

### 2.2. Variables and Instruments

The variables assessed were levels of depression using the CES-D scale, stress hormones (epinephrine, norepinephrine, cortisol, serotonin), hs-CRP, and Igs (IgA, IgG, IgM). All variables were measured twice pre- and post-test in identical conditions.

### 2.3. Physical Characteristics

For physical characteristics, the height (cm), weight (kg), and body mass index (BMI) were measured. DS-102 (Jenix, Seoul, Republic of Korea) was used to measure height and weight, and Inbody720 (Biospace, Seoul, Republic of Korea) was utilized to determine the BMI.

### 2.4. CES-D

CES-D is a depression scale developed by Radloff [16]. The Korean version of CES-D (CES-D for Koreans) with the integrated depression questionnaire that was revised by Chon et al. [17] was used in this study. CES-D is a self-report instrument with which the participant responds based on feelings and activities in the past week. Twenty questions in total were rated on a 4-point Likert scale: 0 “Very rarely (none or one day)”, 1 “Sometimes (one or two days)”, 2 “Often (three to four days)”, and 3 “Almost always (five to seven days)”. An increase in the total score indicated an increase in the level of depression, which was classified as normal (scores 0–15), mild (16–24), and severe (25–60).

### 2.5. Blood Analysis

The variables that required blood collection were stress hormones (dopamine, epinephrine, norepinephrine, cortisol, serotonin), hs-CRP, and Igs (IgA, IgG, IgM). The participants were guided to maintain fasting states (above 8 h) before the analysis. The blood collection was conducted at 8–10 a.m. on the day of the visit; 5 mL of blood was collected from the antecubital vein. Subsequently, the collected blood was centrifuged at 3000 rpm to isolate the serum, which was frozen at ≤70 °C for subsequent analyses.

Catecholamine (dopamine, epinephrine, norepinephrine) was analyzed using the Plasma Catecholamine Kit (Chromsystems, Zug, Switzerland, CHE) and Alliance (Waters, Milford, MA, USA), following the high-performance liquid chromatography method. Cortisol was analyzed using the Cortisol II Kit (Roche, Basel, Switzerland, CHE) and Cobas e801 (Roche, CHE), following the electro-chemiluminescence immunoassay method. Serotonin was analyzed using the Green Cross Kit (Green Cross Corp., Yongin, Republic of Korea, KOR) and 5500 Qtrap (AB SCIEX, Framingham, MA, USA), following the liquid chromatography (LC) method. The serum levels of hs-CRP and Igs (IgA, IgG, and IgM) were analyzed using the Cobas 8000 (Roche, CHE), following the turbidimetric immunoassay method.

### 2.6. Exercise Program

In this study, the walking exercise program comprised common motions. The exercise intensity was identically maintained as in the barre exercise program through the warm-up (10 min), main exercise (30 min with a 5 min rest), and cool-down period (10 min). Using the RS-400sd (Polar, Lake Success, NY, USA), the exercise intensity was set based on the heart rate reserve (HRR) and ratings of perceived exertion (RPE) as follows: 40–50%HRR (RPE 11–12) for 1–4 week, 50–60%HRR (RPE 13–14) for 5–8 week, and 60–70%HRR (RPE 14–15) for 9–12 week. In 4-week intervals, basic motions were added, and their number of repetitions was increased to raise the exercise intensity gradually. And exercise experts conducted the program while checking the individual’s condition and observing the individual’s exercise intensity and posture. Table 2 gives the details of the walking exercise program.

The barre exercise program was based on the program developed by Kim and Oh [15] after revision and modification to suit the purpose of this study. The exercise time was 50 min per session, consisting of the warm-up (10 min), main exercise (30 min), and cool-down (10 min). The exercise was performed thrice a week for a period of 12 weeks. Table 3 presents the details of the barre exercise program.

### 2.7. Statistical Analysis

Using SPSS Version. 27.0 (IBM, Armonk, NY, USA), the collected data were expressed as mean and standard deviation (M ± SD) for the measured variables. Repeated variance analysis measures (ANOVA) were used to test the interaction effects by time and group as well as the simple main effect. A paired *t*-test and one-way ANOVA were used to test the significance of the difference by time and group. Tukey’s test was applied as the post hoc test. The level of significance (*α*) was set at 0.05 for all statistical analyses.

## 3. Results

### 3.1. CES-D

Table 4 presents the results of analyzing CES-D changes in older women after 12-week walking and barre exercise programs.

An interaction effect by time and group was observed in the CES-D scores (*p* < 0.001) with the main effect of time (*p* < 0.001). The BG and WG showed a significant within-group decrease (*p* < 0.05), while the CG showed a significant increase (*p* < 0.05). In the post-exercise test, a significant between-group difference (*p* < 0.01) was observed, and the post hoc test revealed that the depression level was lower in the BG than in the CG.

### 3.2. Stress Hormones

Table 5 presents the results of the assessed changes in stress hormones in older women after 12-week walking and barre exercise programs.

For epinephrine, the main effect of time (*p* < 0.05) was found; the BG showed a significant within-group increase by time (*p* < 0.05). For cortisol, the main effect of time (*p* < 0.05) was observed; the BG and WG showed a significant within-group decrease by time (*p* < 0.05). For serotonin, an interaction effect by time and group (*p* < 0.05) was observed with the main effect of time (*p* < 0.05). The BG (*p* < 0.01) and WG (*p* < 0.05) demonstrated a significant within-group increase by time. In the post-exercise test, a significant between-group difference (*p* < 0.05) was observed, and the post hoc test revealed that the serum level of serotonin was higher in the BG than in the CG.

### 3.3. hs-CRP

Table 6 presents the results of the evaluated and compared changes in hs-CRP in older women after 12-week walking and barre exercise programs. 

An interaction effect by time and group was observed in hs-CRP (*p* < 0.05).

### 3.4. Igs

Table 7 presents the results of changes in Igs in older women after 12-week walking and barre exercise programs. 

For IgA, an interaction effect by time and group (*p* < 0.05) was observed; the BG showed a significant within-group increase (*p* < 0.05). For IgM, an interaction effect by time and group (*p* < 0.01) was observed; the BG showed a significant within-group increase by time (*p* < 0.01).

## 4. Discussion

The effects of a 12-week walking and barre exercise program on CES-D, stress hormones, hs-CRP, and Ig levels in older women were comparatively analyzed, and the results are discussed as follows.

### 4.1. CES-D

Exercise could positively affect the physical and psychological health of older individuals, enhance their health and quality of life, and improve the symptoms of depression [18]. The results of this study demonstrate a decline in CES-D scores in the BG and WG, whereas the level increased in the CG. This result indicates the positive effect of regular physical activity, regardless of the exercise type, in improving depressive symptoms in older women. The increase in CES-D scores in the CG implied a need for an effective intervention to prevent even mild depressive symptoms, as these may lead to severe depression in time.

In previous studies, participation in leisure dancing had a significant effect on positive emotions in older women aged approximately 60 years [19], and the CES-D score was also shown to decrease after outdoor walking exercise in older women [20] and after a health management program including exercise in older adults living at home [21]. These findings are consistent with the results of this study.

Thus, the comparative analysis of the findings of this study and previous studies indicates that a regular exercise intervention, regardless of the type of exercise, is essential for older women to prevent depression.

### 4.2. Stress Hormones

Dopamine is expressed in the brain as a neurotransmitter that plays a vital role in the neural network, with various associations with psychological and emotional characteristics as well as mobility. Physical stimulation, such as exercise, can control energy metabolism and homeostasis while altering the psychological mood. Continuous exercise raises dopamine levels in the pituitary gland [22]. This study demonstrated no significant within- or between-group difference in dopamine. Similarly, in previous studies, the serum level of dopamine did not change in conformity with this study when middle-aged men and women performed a 12-week combined exercise in the morning and afternoon [23] and when middle-aged women performed an 8-week Pilates exercise [24]. Conversely, a previous study reported that dopamine levels increased in older women aged ≥65 years after a 12-week combined exercise [25].

Stress promotes epinephrine secretion by stimulating the sympathetic nervous system. The role of epinephrine is to increase the heart rate and metabolism to supply more blood and oxygen to muscles and to maintain suitable blood pressure levels [26,27]. In this study, the serum level of epinephrine in the BG increased significantly, presumably as part of a positive response mechanism toward rapid adaptation to the external stimulation, which activated neurotransmitters to respond to the physical stress induced by exercise. However, contrasting results were observed in a study on muscle strength training in older women [28] and a study on a combined exercise program in older men and women, where the blood concentration of epinephrine in older women decreased [29].

Norepinephrine is a hormone released in response to an emergency caused by stress. The stimulation by exercise induces a change in norepinephrine, allowing the body to exhibit an efficient response against external stimulation or stress through the ability to regulate physiological and metabolic activities [30]. Neither a change in norepinephrine nor a significant between-group difference was detected in this study. A study that applied muscle strength training to older women reported decreased norepinephrine levels [28]. Another study that applied a 12-week Taekwondo exercise reported no significant change in norepinephrine levels [31]. Consequently, the findings of previous studies both agree and disagree with the findings of this study. The changes in catecholamine levels were thought to be due to complex interactions across participants’ psychological factors and factors such as the type, intensity, and duration of exercise, and the contrasting results were attributed to personal differences in stress response across the participants.

As such, several studies have reported that the role of catecholamine is important in controlling physical function and focus on the importance of exercise time and intensity on sympathetic and adrenal cortex qualities [32].

In this study, cortisol levels decreased significantly in the BG and WG, which could be attributed to the improvement in the stress response to the physical or psychological stimulation of participating in an exercise program. This result is consistent with a study that applied a 12-week Korean traditional dance program with older women [33] and a study that reported a fall in cortisol levels by 18.42% in older women after a 13-week walking exercise program [20]. The similarity between the results of this study and previous research may be attributed to the relationship between changes in the HPA axis and depression in response to stress [34]. We believe that modifications in the HPA axis contribute to positive outcomes in stress relief, facilitated by our exercise program. In general, cortisol concentration is highest in the morning and tends to decrease over time. In the subjects of this study, cortisol was collected when fasting in the morning, and blood was collected with an identical method after 12 weeks. In other words, although blood was collected under the same conditions, the concentration of cortisol decreased afterwards in the two groups who performed exercise, respectively. In addition, since high cortisol concentrations in the morning are very closely related to the occurrence of CVD, it is suggested that the risk of CVD should be considered by observing the increase in cortisol concentration in the morning [35]. This suggests that both the barre group and the walking group in this study decreased in cortisol concentration in the morning after 12 weeks, which could be an effective way to prevent heart disease in elderly women.

Regular exercise or continuous physical activities improve mood disorders, including depression and anxiety, by increasing serotonin expression in the central nervous system [36,37,38]. Serotonin levels in this study increased in the BG and WG. Notably, the serotonin level in the BG was significantly higher than the CG’s. This result is assumed to be due to the natural, positive emotional state, as the participants focused on breathing while performing the motions and the stream of consciousness in the warm-up and cool-down steps of the barre exercise, which repeatedly led to changes in serotonin levels. Previous studies have reported various changes in serotonin expression according to exercise. In a study that applied a 12-week treadmill exercise and another that applied 24-week muscle strength training [28], the serum serotonin levels in older women increased similarly with the results of this study.

Also, regular exercise is reported to induce the secretion of serotonin in the brain through the 5-HT3—IGF-1 mechanism, which is as effective as taking antidepressant drugs, and in the results of this study, we think that the increase in serotonin represents the effect of the exercise program and will have positive effect on antidepressants in elderly women [39]. Additionally, according to [40], old rats have smaller hippocampus, amygdala, and prefrontal cortex than adult rats, and regular exercise increases the number of cells in these tissues to reduce depression and anxiety. Also, serotonin and serotonin A1 receptors can reduce depression-related behavior in old rats. The results of these prior studies support that regular barre and walking exercises in this study positively affected serotonin increases, which can help relieve depression and anxiety in elderly women.

Eventually, we think that special movements such as the intensity of the walking and barre exercises conducted in this study were suitable for elderly women, and we recommend that future studies actively utilize the movements and exercise intensity used in this study.

### 4.3. hs-CRP

An increase in CRP directly affects the pathogenesis of cardiovascular disease in older adults [41]. The level of hs-CRP is associated with reduced mobility in older adults [42]. This study observed an interaction effect by time and group in hs-CRP levels; however, neither a within-group nor between-group difference was observed. Basically, CRP is synthesized by IL-6 in the liver, and these molecules are produced in large quantities when the body becomes susceptible to infection; because of this reason, they are easily found in serum [43].

A study that applied a combined exercise in older women [44] showed no significant difference in hs-CRP levels, similar to the results of this study. Conversely, studies that analyzed the effects of free-weight exercise [45], water- and land-based combined exercise [46], Korean traditional dance [47], and a combined exercise [48] observed a significant decrease in the serum levels of hs-CRP, which is not consistent with the results of this study. Exercise is generally known to reduce hs-CRP levels through anti-inflammatory effects [49]; however, in the case of inflammatory cytokines, factors including diet and oxidative stress are also involved, in addition to exercise [50]. Moreover, the differences in personal health status, age, and sex are thought to be contributing factors.

The decrease in hs-CRP concentration in both exercise groups (WG and BG) in this study is considered physiologically meaningful. These indicate that regular barre exercise may help to maintain the heathy condition of those who suffer from chronic inflammations.

### 4.4. Igs

A reduced level of Igs is associated with aging, while reduced immune functions decrease resistance to disease or external infection. Exercise is a behavioral factor that can enhance immune functions in certain conditions while maintaining or delaying the reduction in Ig functions to serve as a potential adjuvant in immune responses [51,52].

In this study, IgA and IgM displayed an interaction effect by time and group, and the BG showed a significant within-group increase. Such varied results of Ig responses could be due to reduced Ig functions owing to the increased physical stress in older women with a low exercise ability due to the lack of regular exercise in the past, which could have reduced the number or sensitivity of relevant hormones or cellular receptors [53,54]. A mid- to high-intensity exercise was observed to increase the levels of certain Igs, which implies differences in the personal level of exercise. In a previous study that applied an 18-week TaiChi exercise in older women, the exercise group showed a significant increase in IgA and IgG levels [55]. In another study, a 12-week combined exercise comprising walking and resistance exercises was observed to substantially decrease the level of IgG [56]. In a study where older women performed traditional Korean dance, the IgA, IgG, and IgM levels were reported to increase significantly [57]. These findings are both consistent with and differ from the outcomes of this study.

## 5. Limitations and Suggestion

This study has several limitations and suggestions. First, it was difficult to control drugs, diet, sleep pattern, and physical activities in participants’ daily life because we minimized interference in daily life other than our exercise program, which sought to determine whether exercise intervention actually worked in daily life. Second, our study was conducted for a relatively short period of 12 weeks, and it is necessary to additionally confirm the results when exercising (barre) for more than 6 months.

## 6. Conclusions

In conclusion, this study assessed the effects of 12-week walking and barre exercise programs on CES-D, stress hormones, hs-CPR, and Igs in older women.

The results of this study demonstrate that regular walking and barre exercise positively affected CES-D scores and epinephrine, cortisol, and serotonin levels among stress hormones, as well as inflammatory marker hs-CRP in older women. Additionally, the levels of Igs, particularly IgA and IgM, varied significantly.

The results collectively suggest that participation in regular exercise positively affected depression, stress hormones, and immune functions in older women. At the same time, barre, as with walking, was an effective intervention for managing the physical and psychological health of older women.

In particular, not many studies have been conducted on this barre exercise; we think that the effect of the barre exercise has shown great results in relation to stress hormones and immunity functions through this study. These positive results are considered to be the strength of this study. In addition, starting with this study, it is expected that the practical use of the barre movement in elderly women can delay aging and maintain a healthy life.

## Figures and Tables

**Table 1:** Physical characteristics of participants.

Variables	WG (*n* = 9)	BG (*n* = 9)	CG (*n* = 9)
Age (years)	67.33 ± 1.56	67.22 ± 1.31	67.33 ± 1.70
Height (cm)	159.50 ± 4.28	156.81 ± 3.79	156.67 ± 2.92
Weight (kg)	60.42 ± 4.38	57.41 ± 3.51	59.70 ± 5.53
BMI	23.74 ± 1.42	23.32 ± 0.87	24.30 ± 1.73

Values are expressed as M ± SD. WG: walking exercise group; BG: barre exercise group; CG: control group; BMI: body mass index; M: mean; SD: standard deviation.

**Table 2:** Walking exercise program.

Warm-up (10 min)			Stretching (free exercise) and walking in place		
Main exercise (35 min)	1–12 Week	Light and fast walking (5 min rest after 15 min walking)		1–4 Week	40–50% HRR (RPE 11–12)
				5–8 Week	50–60% HRR (RPE 13–14)
				9–12 week	60–70% HRR (RPE 14–15)
Cool-down (5 min)			Walking in place and stretching		

HRR, heart rate reserve; RPE, ratings of perceived exertion.

**Table 3:** Barre exercise program.

Warm-up (10 min)		Floor barre		
Main exercise (30 min)	1–12 Week	Relevé Pull up and Preparation Plié Tendu and Jeté Rond de Jambe a Terre Developpé Grand battement Limbering	1–4 Week	Port de Bras Arabesque, Sissonne
			5–8 Week	Port de Bras, Cambre Small jump
			9–12 Week	Glissade and Assemble Changement
Cool-down (10 min)		Reverence, Body scan		

**Table 4:** Changes in CES-D.

Variables	Group	Pre-Exercise Test	Post-Exercise Test	t		F
CES-D (score)	WG	19.67 ± 2.35	17.11 ± 2.52	4.822 **	Time	30.252 ***
	BG	20.00 ± 2.06	14.67 ± 1.50	5.488 **	Group	2.528
	CG	19.11 ± 2.62	20.33 ± 3.46	−2.475 *	T × G	22.109 ***
	F	0.328	10.582 **			
	Post hoc	NS	BG < CG			

Values are expressed as M ± SD. * *p <* 0.05, ** *p <* 0.01, *** *p <* 0.001. CES-D: Center for Epidemiologic Studies Depression; WG: walking exercise group; BG: barre exercise group; CG: control group; T × G: interaction; M: mean; SD: standard deviation; NS: non-significant.

**Table 5:** Changes in stress hormones.

Variables	Group	Pre-Exercise Test	Post-Exercise Test	t		F
Dopamine (pg/mL)	WG	18.59 ± 2.26	18.74 ± 4.67	−0.122	Time	1.926
	BG	18.27 ± 2.01	19.64 ± 2.39	−1.898	Group	0.232
	CG	18.74 ± 3.38	18.60 ± 2.17	0.195	T × G	0.759
	F	0.078	0.269			
	Post hoc	NS	NS			
Epinephrine (pg/mL)	WG	39.50 ± 12.80	47.61 ± 10.87	−1.832	Time	4.194 *
	BG	41.49 ± 11.11	50.73 ± 16.32	−2.861 *	Group	0.120
	CG	45.18 ± 15.00	44.48 ± 11.73	0.117	T × G	1.344
	F	0.437	0.506			
	Post hoc	NS	NS			
Norepinephrine (pg/mL)	WG	495.48 ± 97.29	523.94 ± 206.07	−0.581	Time	0.491
	BG	609.91 ± 160.10	539.74 ± 206.33	0.711	Group	0.971
	CG	524.42 ± 110.02	485.20 ± 113.18	1.126	T × G	0.572
	F	2.025	0.217			
	Post hoc	NS	NS			
Cortisol (μg/dL)	WG	8.38 ± 2.37	7.47 ± 2.46	3.072 *	Time	4.040 *
	BG	7.88 ± 1.40	6.51 ± 1.93	2.575 *	Group	0.610
	CG	6.95 ± 3.39	6.88 ± 1.13	0.067	T × G	0.958
	F	0.746	0.569			
	Post hoc	NS	NS			
Serotonin (ng/mL)	WG	81.46 ± 35.75	104.94 ± 23.60	−2.818 *	Time	6.808 *
	BG	86.70 ± 25.06	118.19 ± 37.55	−3.667 **	Group	0.955
	CG	90.28 ± 34.71	79.61 ± 23.55	0.885	T × G	5.214 *
	F	0.171	4.115 *			
	Post hoc	NS	BG > CG			

Values are expressed as M ± SD. * *p* < 0.05, ** *p* < 0.01. WG: walking exercise group; BG: barre exercise group; CG: control group; T × G: interaction; M: mean; SD: standard deviation; NS: non-significant.

**Table 6:** Changes in hs-CRP.

Variables	Group	Pre-Exercise Test	Post-Exercise Test	t		F
hs-CRP (mg/L)	WG	0.82 ± 0.56	0.67 ± 0.39	1.405	Time	2.645
	BG	0.94 ± 1.36	0.59 ± 0.85	1.931	Group	0.018
	CG	0.74 ± 0.48	0.88 ± 0.70	−1.512	T × G	3.359 *
	F	0.115	0.441			
	Post hoc	NS	NS			

Values are expressed as M ± SD. * *p* < 0.05. WG: walking exercise group; BG: barre exercise group; CG: control group; T × G: interaction; M: mean; SD: standard deviation; NS: non-significant.

**Table 7:** Changes in Igs.

Variables	Group	Pre-Exercise Test	Post-Exercise Test	t		F
IgA (S) (mg/dL)	WG	236.48 ± 44.23	243.38 ± 46.69	−1.382	Time	1.570
	BG	223.73 ± 44.14	232.02 ± 43.05	−2.788 *	Group	0.217
	CG	228.30 ± 65.87	222.14 ± 57.32	1.437	T × G	3.656 *
	F	0.137	0.417			
IgG (S) (mg/dL)	WG	1146.21 ± 149.78	1260.38 ± 149.44	−1.222	Time	1.359
	BG	1208.21 ± 152.79	1236.86 ± 146.66	−0.716	Group	0.124
	CG	1245.46 ± 179.70	1224.37 ± 195.27	0.885	T × G	1.288
	F	0.869	0.110			
IgM (S) (mg/dL)	WG	103.63 ± 19.51	107.79 ± 16.52	−1.502	Time	1.227
	BG	92.64 ± 29.97	98.93 ± 28.00	−4.274 **	Group	0.853
	CG	113.33 ± 23.96	107.78 ± 27.69	1.779	T × G	6.106 **
	F	1.561	0.387			

Values are expressed as M ± SD. * *p <* 0.05, ** *p <* 0.01. Igs: immunoglobulins; WG: walking exercise group; BG: barre exercise group; CG: control group; T × G: interaction; M: mean; SD: standard deviation.

## Data Availability

The data presented in this study are available on request from the corresponding author.
